# Single-stranded oligonucleotide-mediated in vivo gene repair in the *rd1* retina

**Published:** 2007-05-02

**Authors:** Charlotte Andrieu-Soler, Mounia Halhal, Jeffrey H. Boatright, Staci A. Padove, John M. Nickerson, Eva Stodulkova, Rachael E. Stewart, Vincent T. Ciavatta, Marc Doat, Jean-Claude Jeanny, Therèse de Bizemont, Florian Sennlaub, Yves Courtois, Francine Behar-Cohen

**Affiliations:** 1 Centre de Recherche des Cordeliers, INSERM, U872, Paris, F-75006 France; 2 Université Paris Descartes, UMR S 872, Paris, F-75006 France; 3 Université Pierre et Marie Curie- Paris 6, UMR S 872, Paris, F-75006 France; 4 Optis France, Paris, 75015, France; 5 Emory University, Department of Ophthalmology, Atlanta, GA; 6 Rothschild Ophthalmologic Foundation, Paris, France

## Abstract

**Purpose:**

The aim of this study was to test whether oligonucleotide-targeted gene repair can correct the point mutation in genomic DNA of PDE6b*^rd1^* (*rd1*) mouse retinas in vivo.

**Methods:**

Oligonucleotides (ODNs) of 25 nucleotide length and complementary to genomic sequence subsuming the *rd1* point mutation in the gene encoding the β-subunit of rod photoreceptor cGMP-phosphodiesterase (β-PDE), were synthesized with a wild type nucleotide base at the *rd1* point mutation position. Control ODNs contained the same nucleotide bases as the wild type ODNs but with varying degrees of sequence mismatch. We previously developed a repeatable and relatively non-invasive technique to enhance ODN delivery to photoreceptor nuclei using transpalpebral iontophoresis prior to intravitreal ODN injection. Three such treatments were performed on C3H/henJ (*rd1*) mouse pups before postnatal day (PN) 9. Treatment outcomes were evaluated at PN28 or PN33, when retinal degeneration was nearly complete in the untreated *rd1* mice. The effect of treatment on photoreceptor survival was evaluated by counting the number of nuclei of photoreceptor cells and by assessing rhodopsin immunohistochemistry on flat-mount retinas and sections. Gene repair in the retina was quantified by allele-specific real time PCR and by detection of β-PDE-immunoreactive photoreceptors. Confirmatory experiments were conducted using independent *rd1* colonies in separate laboratories. These experiments had an additional negative control ODN that contained the *rd1* mutant nucleotide base at the *rd1* point mutation site such that the sole difference between treatment with wild type and control ODN was the single base at the *rd1* point mutation site.

**Results:**

Iontophoresis enhanced the penetration of intravitreally injected ODNs in all retinal layers. Using this delivery technique, significant survival of photoreceptors was observed in retinas from eyes treated with wild type ODNs but not control ODNs as demonstrated by cell counting and rhodopsin immunoreactivity at PN28. β-PDE immunoreactivity was present in retinas from eyes treated with wild type ODN but not from those treated with control ODNs. Gene correction demonstrated by allele-specific real time PCR and by counts of β-PDE-immunoreactive cells was estimated at 0.2%. Independent confirmatory experiments showed that retinas from eyes treated with wild type ODN contained many more rhodopsin immunoreactive cells compared to retinas treated with control (*rd1* sequence) ODN, even when harvested at PN33.

**Conclusions:**

Short ODNs can be delivered with repeatable efficiency to mouse photoreceptor cells in vivo using a combination of intravitreal injection and iontophoresis. Delivery of therapeutic ODNs to *rd1* mouse eyes resulted in genomic DNA conversion from mutant to wild type sequence, low but observable β-PDE immunoreactivity, and preservation of rhodopsin immunopositive cells in the outer nuclear layer, suggesting that ODN-directed gene repair occurred and preserved rod photoreceptor cells. Effects were not seen in eyes treated with buffer or with ODNs having the *rd1* mutant sequence, a definitive control for this therapeutic approach. Importantly, critical experiments were confirmed in two laboratories by several different researchers using independent mouse colonies and ODN preparations from separate sources. These findings suggest that targeted gene repair can be achieved in the retina following enhanced ODN delivery.

## Introduction

The PDE6b*^rd1^* (*rd1*) mouse is a model of rapid retinal degeneration [1]. It results from a point mutation in the gene encoding the β-subunit of rod photoreceptor cGMP-phosphodiesterase (β-PDE), leading to a stop codon (Tyr347Ter), truncated protein, and nonsense-mediated mRNA decay [2]. In the *rd1* mouse, rod photoreceptor loss is nearly complete by postnatal day (PN) 21 [3,4]. Mutations in the same gene are responsible for retinal degeneration in patients with retinitis pigmentosa [5,6].

Targeted gene repair aims to correct mutations in genomic DNA by using RNA/DNA oligonucleotides (RDOs) or single-stranded DNA oligonucleotides (ssODNs) [7,8]. This gene therapy strategy should allow for a permanent correction of the genomic DNA and for normal physiologic regulation of the corrected gene by its endogenous promoter [7,8]. Targeted gene repair has been effective in inducing genotypic and phenotypic corrections both in vitro and in several animal models of various disorders such as hemophilia, Crigler-Najjar syndrome type 1, albinism, Duchenne muscular dystrophy, hyperlipidemia type 2, and sickle cell disease [9-22]. The majority of in vivo studies have used RDOs. Recently, successful repair has been described in vivo with phosphorothioate single-stranded ssODNs [23-25]. Compared to RDOs, ssODNs present advantages: (1) Their synthesis is more efficient, with higher yields and purity; (2) they are less expensive; and (3) they are more stable. Moreover, the induced repair is more reproducible [26]. Reproducibility is one of the major limitations of gene repair using RDOs, so enhanced reproducibility with ssODNs is of considerable importance [27].

Efficient DNA delivery to photoreceptor nuclei is requisite for targeted gene repair to occur [28,29]. Within the living eye, effective gene therapy relies on high transfection efficiency of the cells of interest [30]. Subretinal or intravitreal injection does not ensure efficient transfection of photoreceptor cells. We recently demonstrated that the *rd1* mutation can be corrected in vitro by gene repair in non-ocular cell lines using locked nucleic acids (LNAs) and phosphorothioate ODNs designed to correct the point mutation in the β-PDE gene [25]. To translate these results to the *rd1* mouse in vivo, we evaluated the effect of current to enhance the delivery of ODNs to photoreceptors [29]. Low current density iontophoresis indeed safely promotes intraocular penetration of drugs [31-34] and gene fragments [35-37]. We also previously observed that iontophoresis enhances the intracellular penetration of intact ODNs in corneal cells [35]. Preliminary in vivo findings using this iontophoresis procedure to deliver phosphorothioate ODN and LNA ODNs in the *rd1* mouse retina have shown beneficial effects on photoreceptors survival [25].

In the work reported here, we evaluated whether iontophoresis performed immediately before the intravitreal injection of ODNs enhanced their localization into mouse photoreceptor nuclei and resulted in gene repair and altered phenotype. We report that delivery of specific phosphorothioate ODNs designed to correct the point mutation in the β-PDE gene, using iontophoresis coupled to intravitreal injection of ODNs, induced genotypic and phenotypic changes of the *rd1* retina. Conversion of the mutant nucleotide to wild type in this model is associated with appearance of β-PDE immunoreactivity in retinal cells, partial preservation of rhodopsin immunoreactive cells in the outer nuclear layer (ONL), and increased photoreceptor cell counts. Our genotypic and phenotypic data show that, though modest, targeted gene repair was achieved in vivo in the *rd1* mouse neural retina.

## Methods

### Animals

C3H/HenJ mice homozygous for the nonsense mutation (amino acid position 347) in the β-PDE gene were used. Wild type mice (C57BL6 or 129sv) served as positive controls. Mice were obtained from Janvier (Le Genest, France) and The Jackson Laboratory (Bar Harbor, Maine). Mice were maintained in clear plastic cages and subjected to a standard 12h:12h light-dark cycle. Experiments were conducted in accordance with the ARVO Statement for the Use of Animals in Ophthalmologic and Vision Research and the institutional guidelines regarding animal experimentation in Ophthalmic and Vision Research.

### Oligonucleotides

ODNs of 25-nucleotide length and with sequence subsuming the *rd1* point mutation were synthesized and purified by high pressure liquid chromatography (Proligo, Paris, France). ODNs were synthesized with six phosphorothioate linkages at 5' and 3' ends (Table 1). ODNs in distilled water were quantified by absorbance at 260 nm. The sense (S) and antisense (AS) wild type (WT) alleles of the β-PDE gene sequence were synthesized (WTS and WTAS, respectively; see Table 1 for nomenclature and sequences). Other ODNs were synthesized with the same bases as WTS, but with some or all out of order (e.g., containing mismatches to the genomic sequence). The central seven bases of WTSscr7 and all 25 bases of WTSscr25 were scrambled (Table 1). These ODNs were not expected to induce gene repair and were thus used as negative controls. One preparation of WTS was 5' labeled with CY3 for use in localization studies.

**Table 1:** Sequence of the oligonucleotides tested for in vivo gene repair.

ODN name	Sequence
WTS	C*C*T*T*C*C*AACCTACGTAGCA*G*A*A*A*G*T
WTAS	A*C*T*T*T*C*TGCTACGTAGGTT*G*G*A*A*G*G
WTSscr7	C*C*T*T*C*C*AACAACGTCTGCA*G*A*A*A*G*T
WTSscr25	A*A*T*C*A*C*AGTTGCCTATAGG*A*C*C*C*C*A
rd1S	C*C*T*T*C*C*AACCTAAGTAGCA*G*A*A*A*G*T

These oligonucleotides (ODNs) were used in the gene repair experiments detailed in the text. Sequences are presented 5' to 3', left to right. "WTS" is wild type sense sequence and "WTAS" is wild type antisense sequence. Treatment with these two ODNs is hypothesized to lead to conversion of the *rd1* mutation to wild type in genomic DNA. "WTSscr7" is wild type sense sequence at the 5' and 3' ends, but the middle seven bases, though the same as those in wild type, are out of order, or scrambled (i.e., containing mismatches to the genomic sequence). "WTSscr25" contains the same bases as WTS but with all bases out of order compared to wild type sequence. "rd1S" is *rd1* sense sequence. That is, it contains the *rd1* point mutation, an adenine, rather than the wild type cytosine, at the 13^th^ position, but is otherwise identical to WTS. The last three ODNs should not be able to induce genomic base conversion. Asterisk (*) represents phoshphorothiate linkages, which were used to prevent nuclease degradation.

### Iontophoresis and injection

A transpalpebral (across eyelids) iontophoresis system was used (patent number FR2830766). We have found that applying transpalpebral iontophoresis immediately after or before intravitreal injection of ODNs leads to the same penetration efficiencies [38]. Therefore, we chose to perform iontophoresis immediately prior to the intravitreal injection of ODNs in order to avoid manipulation of the injected pups' eyes and reduce the potential danger of secondary infection.

**Figure 1:**
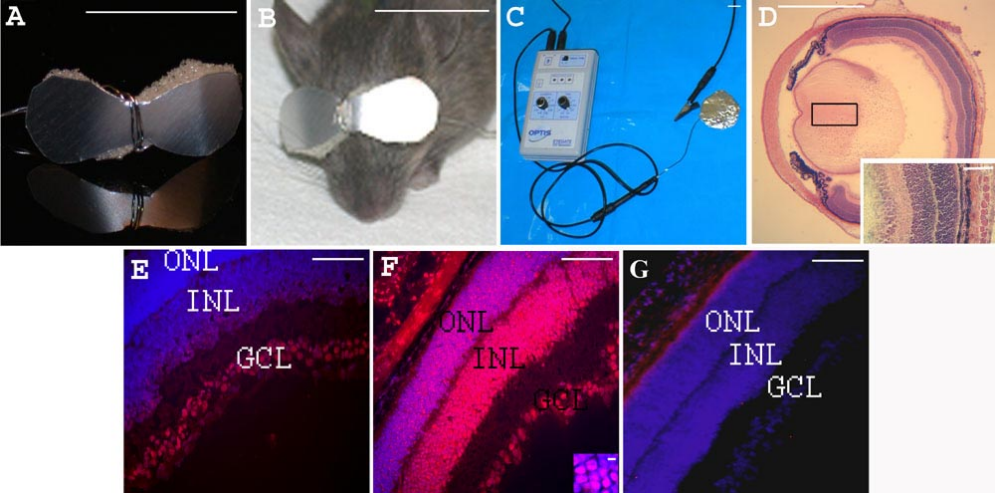
Iontophoresis device and eye sections from PN7 *rd1* mice, 1 h after treatment. Iontophoresis device. **A**: An eye-glass-shaped electrode was made with aluminum foil and single-use disposable medical grade hydrophilic polyurethane sponge. **B**: The electrode covered both closed eyelids of the treated newborn mouse iontophoresis. **C**: shows the generator and the return electrode. Eye section 1 h after transpalpebral iontophoresis. **D**: Hematoxylin and eosin stained eye section showing integrity of the eye structures after iontophoresis Inset shows tissue at high magnification. Eye sections 1 h after intravitreal injection of CY3-tagged oligonucleotide. In E-G, nuclei were stained blue with DAPI and red with CY3. **E**: without prior iontophoresis, **F**: with prior iontophoresis (inset: high magnification of the ONL). **G**: Control retina from an *rd1* mouse injected with 1 μl of PBS with prior iontophoresis. The following abbreviations were used: outer nuclear layer (ONL), inner nuclear layer (INL), and ganglion cell layer (GCL). Scale bars: **A**, **B**, **C**, 1 cm; **D** 1 mm; **E**, **F**, **G** and inset **D**, 100 μm; inset **F**, 5 μm.

Prior to iontophoresis, pups' eyelids were opened with a scalpel (Swann Morton, Peynier, France) if needed and tetracaine 1% drops (Novartis Ophthalmics SA, Rueil Malmaison, France) were instilled. An hour-glass-shaped aluminum foil and disposable medical grade hydrophilic polyurethane sponge (3.2 mm thick, 1.5x0.7 cm length by width; Optis, Levallois, France), was soaked in phosphate buffered saline (PBS: 0.2 g/l KCl, 0.2 g/l KH_2_PO_4_, 8 g/l NaCl, 2.16 g/l Na_2_HPO_4_ 7H_2_O, pH 7.4) and used as the active negative electrode (Figure 1A). The electrode covered both eyelids and was connected to the generator with the clip shown at the top of Figure 1C. The return electrode was connected to the tail and hind foot pads of the mouse. Anionic iontophoresis (negative electrode connected to the eyelids) was performed with a 1.5 mA current for 5 min (1.43 mA/cm^2^; Figure 1B). An audio-visual alarm indicated any disruption of the electric circuit ensuring a controlled delivery of the current.

Intraocular injections were carried out with borosilicate micropipette needles (Phymep, Paris, France) pulled with a pipette puller (model 720, Kopf Instruments,Tujunga, California) and cut at 2 mm from the neck, leading to a 60 μm injection outer diameter. Micropipette needles were linked to an Eppendorf microinjector 5242 (Roucaire, Velizy, France). 1 μl of PBS or ODN (concentrations are given in the next section) was injected into the vitreous. The position of the needle was monitored by observation under a dissecting microscope through a glass cover slip placed on the corneal surface. To limit loss of the injected solution and allow the intraocular pressure to equilibrate (as observed by the return of normal iris perfusion), the micropipette needle was left in place for 10 s before withdrawal.

For tissue harvest, mice were sacrificed by a lethal dose of pentobarbital (6 g/100 ml; Ceva Santé Animale, Libourne, France) injected intraperitoneally.

### Localization of oligonucleotides

Eight eyes of *rd1* mice at PN7 underwent a single transpalpebral iontophoresis (anionic, 5 min, 1.5 mA) followed by an intravitreal injection of 1 μl of CY3-labeled WTS ODN (1 μl of 272 μM). For the control groups, *rd1* mice at PN7 received either iontophoresis followed by PBS injection, ODN injection without iontophoresis, iontophoresis without injection, or had no treatment (8 eyes for each condition). Animals were sacrificed 1 h after treatment. The eyes were enucleated, rinsed in PBS, and embedded with Tissue-Tek OCT-compound (Bayer Diagnostics, Puteaux, France) for cryo-sectioning. Sections (10 μm) were fixed in 4% paraformaldehyde (Merck Eurolab, Strasbourg, France) for 5 min at room temperature, washed in PBS, counter-stained for 2 min with DAPI (4',6-diamino-2-phenylindole; 1/3000 dilution; Sigma-Aldrich, Saint-Quentin Fallavier, France), washed in PBS, mounted in Gel Mount (Microm Microtech, Francheville, France) and examined under a fluorescence microscope (Aristoplan, Leica, Rueil Malmaison, France) with HBO103w lamp and a digital SPOT camera (Optilas, Evry, France). For each eye, sections at the optic nerve level were counter-stained with hematoxylin and eosin (H&E) for structural analysis.

### Outer nuclear layer cell counting

Eyes of PN28 mice were enucleated, quickly frozen in Tissue-Tek OCT-compound (Bayer Diagnostics), and sectioned (10 mm). For each eye, 5 sections that included optic nerve were H&E stained. For each section, the number of nuclei in the ONL was counted in the same region at 400 mm from each edge of the optic nerve over a 400 mm length (n=10 values for each eye). Each section was counted by at least two observers. Observers did not know which experimental group sections were from (i.e., counts were conducted in a double-blind manner).

### Rhodopsin immunohistochemistry on whole flat-mount retinas

Rhodopsin immunohistochemistry was assessed on whole flat-mount retinas as previously described [39]. Briefly, at PN19 and 28, ocular globes were fixed in 4% paraformaldehyde (Merck Eurolab) for 1 h. Retinas were isolated, placed in PBS in 1.5 ml microcentrifuge tubes, permeabilized in PBS, 0.1% Triton X-100 (Sigma-Aldrich) for 5 min, and incubated in blocking buffer (PBS containing 0.1% bovine serum albumin, 0.1% Tween 20 and 0.1% sodium azide; all chemicals from Sigma-Aldrich) for 15 min. Retinas were incubated for one hour with the rhodopsin-specific mouse monoclonal antibody, rho-4D2 (1/100 dilution in blocking buffer; kindly provided by Dr. Robert Molday, University of British Columbia, Vancouver BC, Canada [40]). As negative controls, normal mouse serum (Nordic Immunological Laboratories, Tebu-bio, Le Perray en Yvelines, France) or mouse monoclonal antibody Leu-M5 directed against macrophages and monocytes (BD Biosciences, Pont-de-Claix, France) were used instead of rho-4D2 antibody (1/100 dilution in blocking buffer, 1 h). Then, the retinas were washed three times for 5 min in blocking buffer and incubated with a secondary goat anti-IgG mouse antibody conjugated to Alexa Fluor 488 (1/250 dilution in blocking buffer; Molecular Probes, Leiden, the Netherlands). The incubation volumes were 0.2 ml for antibody incubations and 1.5 ml for blocking and washing steps. After washing three times in PBS for 5 min, retinas were mounted in PBS-glycerol (1/1), with the photoreceptor layer facing up, and examined by fluorescence microscopy with a 2.5X objective and photographed using a digital SPOT camera (Optilas). All pictures were taken with an exposure time of 5 s. For each retina, three pictures were taken to cover the whole retinal surface. Photographs of flat-mounts were merged in Photoshop 7.0 (Adobe Systems Inc., San Jose, California) to reconstruct the whole retina.

Intensity of rhodopsin immunoreactivity was quantified by using the luminosity feature of Photoshop for raw pictures. Tissue and background regions were manually selected. Any residual pigmented epithelium was excluded. Mean pixel brightness was determined for each region by using the "Histogram" imaging feature. To normalize background levels among images, the mean brightness level per pixel of the tissue region was divided by the background region from each flat-mount image.

Experiments with the WTS ODN, the corresponding two negative control ODNs (WTSscr7 and WTSscr25), and the untreated or PBS-treated controls were repeated three more times (six eyes for each condition, three additional, replicated experiments). Other controls included injection of the WTS ODN without iontophoresis and iontophoresis without any injection (4 eyes for each condition).

The effect of the number of treatments on response was assessed by injecting WTS ODN (1 μl of 500 μM) following iontophoresis at PN4 (one injection total), at PN4 and PN6 (two injections total), at PN4, 6, and 8 (three injections total), or at PN6, PN8 and PN10 (three injections total). Each condition was tested on four eyes.

### Rhodopsin immunohistochemistry on PN28 eye sections

Eyes of PN28 PBS-treated (six eyes) or ODN-treated (eight eyes) *rd1* mice and untreated wild type (four eyes) were enucleated, quickly frozen in Tissue-Tek OCT-compound (Bayer Diagnostics), and sectioned (10 mm). For each eye, sections that included optic nerve were H&E stained with for structural analysis. Sections were fixed in 4% paraformaldehyde (Merck Eurolab) for 5 min at room temperature, washed in PBS, and incubated 1 h in mouse rho-4D2 antibody (1/100 dilution in PBS). As negative controls, normal mouse serum (Nordic Immunological Laboratories) or mouse monoclonal antibody Leu-M5 (BD Biosciences) replaced the primary antibodies (1/100 dilution in PBS). Slides were washed three times in PBS and incubated with mouse anti-IgG conjugated to Alexa Fluor 488 (1/250 dilution in PBS; Molecular Probes). Then, the slides were washed three times in PBS, mounted in PBS/glycerol (1/1) and examined under a fluorescence microscope (Leica).

### β-phosphodiesterase immunohistochemistry on eye sections

The presence of β-PDE immunoreactivity was assessed in eye sections from PN28 untreated and PBS- or ODN-treated *rd1* mice previously labeled with rho-4D2. Immunohistochemistry was performed with rabbit IgG PDE6b antibody (1/100 dilution; Affinity Bioreagents, Golden, Colorado) or non-immune rabbit serum (1/100 dilution) as primary antibodies and goat anti-IgG rabbit antibody conjugated to Texas Red Fluor (1/100 dilution; Molecular Probes) as secondary antibody. Sections were mounted in PBS/glycerol (1/1) and examined under a fluorescence microscope (Leica). Primary and secondary antibody concentrations and incubation times were optimized such that background signal was undetectable in untreated *rd1* retina sections or when normal serum was substituted for primary antibody [41] (see Results section). The specificity and selectivity of the primary antibody was demonstrated by the observations that (1) only photoreceptor cells showed immunoreactivity in wild type retina sections and (2) only protein extracts from wild type retinas, but not *rd1* retinas, showed immunoreactivity with bands of correct size in an immunoblot ([41] and Results section).

### Genotypic changes induced in *rd1* retinas treated with WTS

Genomic DNA from *rd1* retinas of mice treated with WTS was extracted from individual whole flat-mount retinas (PN28) using a DNeasy Tissue kit and eluted in 200 μl of AE buffer as per manufacturer's instructions (Qiagen, Courtaboeuf, France and Valencia, California). Genomic DNA from wild type retinas, untreated, and PBS-treated *rd1* retinas served as controls.

Allele-specific real-time PCR was used to detect small amounts of wild type sequence resulting from ODN treatment. DNA samples isolated from treated and untreated retinas were used as template DNA in PCR reactions with primers designed to preferentially amplify wild type rather than mutant β-PDE sequence. The 3' base of one primer was complementary to wild type sequence, but not *rd1* sequence, at position 1048 of GenBank accession number X60133 [42]. Primers used for preferential amplification of wild type β-PDE sequence were 5'-TGC AAG CAT TCA TTC CTT CGA C-3' and 5'-AAG CCA CTT TCT GCT ACG-3'. For normalization calculations, parallel reactions using aliquots of the same source of template DNA were run using primers designed to amplify both wild type and *rd1* mutant β-PDE sequences (W149 and W150 of reference [4]): 5'-CAT CCC ACC TGA GCT CAC AGA AAG-3' and 5'-GCC TAC AAC AGA GGA GCT TCT AGC-3'. Reactions were run in a Bio-Rad iCycler iQ real time PCR detection system with melt curve analysis (Bio-Rad, Hercules, CA). Reactions of 20 μl final volume included template DNA (10 ng), primers (50 nM), and QuantiTect SYBR Green PCR Master Mix (Qiagen, Valencia, CA), which is composed of SYBR Green I (a dye that fluoresces strongly when bound to dsDNA), HotStarTaq DNA polymerase, dNTPs, and buffer components optimized by the manufacturer. Poly (dI:dC), 20 ng per assay, was added to reduce nonspecific PCR products.

The limit of detection for quantification was considered 10 times the root mean square noise of fluorescence intensity across a window usually spanning cycles 2 through 10. The cycle number at which product accumulated past this detection threshold (C_t_) was related to beginning copy number of a specific template allele in a reaction by a calibration curve created with standard amounts of the wild type β-PDE gene. A lower C_t_ compared to untreated *rd1* controls indicates the presence of a specific allele, in this case, a presumed *rd1* allele repaired to wild type sequence (mutant adenine converted to wild type cytosine). For each template DNA, the C_t_ from either mutant or wild type allele specific β-PDE reactions were subtracted from C_t_ of a non-allele specific reaction to correct for differences in total DNA starting concentrations. Normalizing reactions were identical to β-PDE reactions with the exception that PCR primers specific to the 18S-RNA gene were used to amplify template DNA. C_t_ data are means±SEM of 5-6 experimental samples assayed in 5-10 replicates. The efficiency of the assay was determined by making calibration curves of gene copy versus threshold cycle were made using increasing amounts of wild type genomic DNA (1 pg to 10 ng) mixed with 10 ng of *rd1* genomic DNA.

In separate control assays, various repair ODNs that contained wild type sequence were added into DNA template samples at several concentrations to determine whether their presence caused artificial decreases or increases in C_t_s. Their presence had no effect on C_t_s (data not shown) over a wide range of concentrations.

### Statistical analysis

Unless otherwise noted, results were expressed as means±SD and compared using the non-parametric Mann-Whitney test and analysis of variance (ANOVA) with post-hoc Student-Newman-Keuls test. p<0.05 was considered as significant.

### Confirmation of phenotypic changes following oligonucleotide treatment

Confirmatory experiments were conducted independently in collaborators' laboratories using the methodology described above and previously published [38] with a few exceptions. Mice for experiments were bred from C3H/henJ mice obtained from The Jackson Laboratory (Bar Harbor, ME, USA) and genotyped to confirm the homozygous presence of the *rd1* point mutation using the strategy of Pittler and Baehr [4] (data not shown). Pups were treated with either WTS or rd1S ODNs (Table 1) that were synthesized and purified by the Emory University Microchemical Facility (Atlanta, Georgia). Littermates were always used and both ODNs were always tested preclude handling or rearing confounds. In some experiments, both eyes of a mouse received either WTS or rd1S. In other experiments, each mouse received WTS in one eye and rd1S in the other eye.

ODNs were delivered as described above with the exception that eyelids were always slit open prior to iontophoresis. The local anesthetic used was 0.5% proparacaine (Akorn Inc., Buffalo Grove, Illinois), the polyurethane foam was 1.6 to 3.2 mm thick (Rynel, Wiscasset, Maine), and 0.5 μl of a 500 μM ODN solution was intravitreally injected. Following each of the three treatments, Refresh Tears (Allergan, Inc.; Irvine, CA, USA) and Tribiotic Ointment (Taro Pharmaceuticals, Bramalea, Ontario, Canada) were applied to eyes and eyelids.

Treated pups were sacrificed at PN33 by carbon dioxide inhalation. Eyes were harvested and prepared for immunohistology substantially as previously published [43]. Eyes were marked on the superior limbus with indelible ink while still in the socket for orientation. They were removed with forceps, then injected at the ink mark with approximately 1 μl of 10% buffered formalin (Stephens Scientific, Riverdale, New Jersey). Eyes were soaked in formalin for 30 min, rinsed in PBS, then stored in PBS in microcentrifuge tubes at 4 °C awaiting further processing. For immunohistochemical analysis, eyes were dehydrated through a graded series of alcohol and xylene then embedded in paraffin wax using a Histocentre 2 embedding center (Thermo Shandon, Waltham, MA). Sections (5 μm) bisecting the optic disc superiorly to inferiorly were cut on an American Optical/Spencer microtome (Buffalo, NY) and fixed to glass slides.

Paraffin-embedded retina sections were deparaffinized, permeabilized in 0.1%Triton X-100/PBS, blocked with 10% normal goat serum in SuperBlock Buffer in PBS with 0.05% Tween-20 added for 30 min, incubated with one of two primary antibodies specific to rhodopsin (1D4 or 4D2, gifts of Dr. Robert Molday [40,44]; 1 μg/ml) for 1 h, then incubated with Oregon Green-conjugated goat anti-rabbit IgG (1:1000 dilution; Molecular Probes, Eugene, OR, USA), and counter-stained with propidium iodide (Molecular Probes), a drop of SlowFade Light antifade medium was put on the section and a cover slip placed on the slide. Sections were then observed and photographed by computer-aided confocal microscopy (Optiphot 2 microscope, Nikon Corporation, Melville, NY; Bio-Rad MRC 1024 using filters 585 EFLP (for PI) and 522 DF32 (for fluorescein (488)), argon-krypton laser, running LaserSharp 2000 version 5.2 build 824, Bio-Rad, Hercules, CA). Images of individual fields were combined to produce images of entire retinal sections using Adobe Photoshop CS (version 8.0, Adobe Systems, Inc.). Cells of the ONL that stained positive for rhodopsin immunoreactivity were counted using Image Tool (UTHSCSA, San Antonio, Texas).

One to three sections were assessed for each eye. Immunopositive cells in the ONL of each image were counted by three observers who were unaware of the experimental group from which the image originated. Observers' counts were averaged for each section. Some sections were missing segments of tissue due to preparation problems (e.g., microtome blade chatter). This loss could artificially lower immunopositive cells counts. To compensate for such artifacts, the length of tissue from which counts were actually derived was measured. The immunopositive cell count mean was divided by this length and the result was used as the sample value for that particular section. Group data are reported as mean immunopositive cell number/cm tissue length ±SEM.

An unpaired Student's t-test was used to compare means of rd1S- versus WTS-treated groups in experiments in which both eyes of a mouse were treated with the same ODN. In experiments in which each mouse received WTS in one eye and rd1S in the other eye, mean differences between treatment groups were assessed using a paired Student's t-test.

## Results

### Delivery of oligonucleotides into retinal cells

Transpalpebral iontophoresis was performed on PN7 mice using an hour-glass-shaped electrode made with aluminum foil and surgical sponge (Figure 1A) and connected to a power supply (Figure 1B). Iontophoresis did not cause any detectable gross or histologic lesions to mouse eyes (Figure 1D and [38]). When CY3-labeled WTS ODN was intravitreally injected, but no inotophoresis was performed, fluorescence was observed in the ganglion cell layer (GCL) and in a few cells within the inner nuclear layer (INL) but not in the ONL (Figure 1E). In contrast, when PBS iontophoresis was applied immediately prior to the ODN injection, intense fluorescence was observed both in nuclei of the INL and the ONL (Figure 1F). No fluorescence was observed in any retinal layers of PBS-treated eyes (Figure 1G) or uninjected control eyes (with or without iontophoresis; data not shown; 8 eyes for each condition).

Independent, confirmatory experiments similarly showed ODN delivery to all retinal neural layers. No difference was observed between iontophoresis being given before or after intravitreal injection of fluorescently-labeled ODN, similar to results previously published [38] (data not shown).

### Outer nuclear layer nuclei numbers at PN28 are preserved following treatment specifically with wild type oligonucleotides

**Figure 2:**
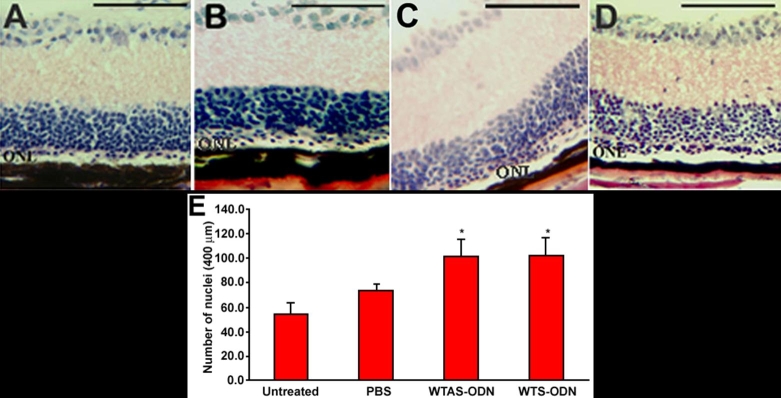
Eye sections of treated and control PN28 *rd1* mice and outer nuclear layer cell counting. Hematoxylin and eosin stained sections of *rd1* eyes showed an increased number of nuclei rows in the outer nuclear layer (ONL) of oligonucleotide (ODN)-treated eyes. **A**: Untreated mouse. **B**: PBS-treated mouse. **C**: Mouse treated with WTAS ODN (corresponding to wild type antisense sequence). **D**: Mouse treated with WTS ODN (corresponding to wild type sense sequence). **E**: Counting of nuclei in the ONL shows a significant increase of nuclei in WTAS ODN- and WTS ODN-treated eyes compared to PBS-treated and untreated eyes (*p<0.05). Scale bars are **A**, **B**, **C**, and **D**, 100 μm.

At PN4, PN6, and PN8, *rd1* mice received iontophoresis then were intravitreally injected with PBS vehicle-only or the various ODNs (1 μl of 500 μM solution). At PN28, mice were sacrificed, eye enucleated, retina sections prepared, and nuclei of the ONL counted. While a single row or less of sparse cells was observed at PN28 in the ONL of untreated *rd1* retina (Figure 2A), discontinuous areas containing two or three rows of nuclei were observed over the ONL of mice treated with WTAS ODN and WTS ODN. Figure 2C,D represent two of those areas where maximal rescue was observed. In PBS-treated eyes, a smaller increase was observed in the ONL (Figure 2B).

Quantification of these observations shows that treatment with wild type ODNs resulted in the preservation nuclei in the ONL. ONL cell counts following treatment with either WTAS (101±15, mean±SD, on a 400 mm length; 8 eyes) or WTS (103±14; 8 eyes) were significantly increased compared to the number from PBS-treated retinas (74±5; 6 eyes) or untreated retinas from *rd1* mice (55±8; 6 eyes; p<0.01; simple ANOVA with Student-Newman-Kuels post-hoc testing). The number of nuclei in the ONL was not significantly different in retinas treated with WTAS versus WTS (p>0.05). The number of nuclei in the ONL was not significantly different in untreated retinas versus PBS-treated retinas (p>0.05).

### Rhodopsin immunostaining at PN28 is increased following treatment specifically with wild type oligonucleotides

Rhodopsin is the most abundantly-expressed photoreceptor-specific protein and is frequently used as a marker to detect the existence of rod photoreceptor cells [45-47]. To evaluate the potential of ODNs to induce gene correction and subsequent photoreceptor survival, rhodopsin immunohistochemistry was performed on retina preparations from treated *rd1* retinas at PN28. As in previous experiments, *rd1* mice were subjected to iontophoresis followed by injection with 1 μl of 500 μM WTS ODN at PN4, PN6, and PN8, with subsequent sacrifice and tissue preparation at PN28. Wild type tissue was examined at PN19 and PN28.

**Figure 3:**
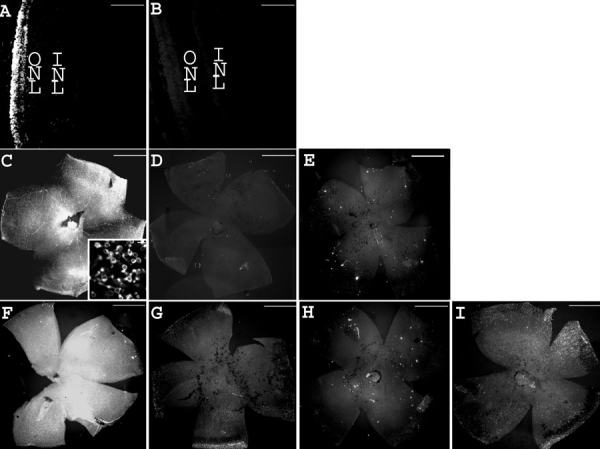
Treatment with WTS preserves rhodopsin at PN28. Rhodopsin immunohistochemistry on wild type eye sections and *rd1* whole flat-mount retinas, reflecting the time course of the retinal degeneration and the treatment efficacy. **A**: Wild type eye section from a mouse at PN28. **B**: Control eye section from wild type mouse at PN28 using normal mouse serum. **C**: *rd1* flat-mount retina from a mouse at PN19 (inset: high magnification). **D**: Control flat-mount retina from *rd1* mouse at PN19 using normal mouse serum. **E**: *rd1* flat-mount retina from a mouse at PN28. **F**: PN28 *rd1* flat-mount retina injected by WTS with prior iontophoresis at PN4, PN6, and PN8. **G**: PN28 *rd1* flat-mount retina injected by WTS without prior iontophoresis at PN4, PN6, and PN8. **H**: PN28 *rd1* flat-mount retina iontophoresed without oligonucleotide injection at PN4, PN6, and PN8. **I**: PN28 *rd1* flat-mount retina injected with WTSscr7 with prior iontophoresis at PN4, PN6, and PN8. Scale bars are **A** and **B**, 100 μm; **C**, **D**, **E**, **F**, **G**, **H**, and **I**, 1 mm; inset, 10 μm.

Extensive positive immunoreactive signal for rhodopsin was observed in wild type eye sections at PN28 (Figure 3A). Sections reacted with normal mouse serum in place of rho-4D2 yielded no signal over background (Figure 3B). In *rd1* flat-mount retinas, rhodopsin-positive signal was observed at PN19 (Figure 3C), consistent with incomplete retinal degeneration at this age. The intense fluorescence observed at low magnification at PN19 corresponded to dispersed immunoreactive photoreceptors as shown at a higher magnification (inset in Figure 3C). No positive signal was detected when normal mouse serum was used as control on *rd1* flat-mount retinas at PN19 (Figure 3D). At PN28, the rhodopsin signal was extremely low, reflecting the advanced and nearly complete degeneration of rods in *rd1* retinas at this time point (Figure 3E), paralleling the well-established time course of the *rd1* retinal degeneration [45-47].

Conversely, as quantified in Table 2 and exemplified in Figure 3F, treatment with WTS ODN yielded rhodopsin immunostaining that was more intense compared to no treatment, treatment with PBS alone, or treatment with either of the negative control ODNs containing sequence mismatches (WTSscr25, and WTSscr7). Further, treatment with negative control ODNs did not induce significant increases in rhodopsin immunostaining compared to PBS treatment (Figure 3I and Table 2), demonstrating the specificity of the response to the combined iontophoresis/injection treatment with WTS ODN. When WTS ODN injection was not coupled to the application of current (Figure 3G) or when the current was applied without any intravitreal injection (Figure 3H), no effect on rhodopsin immunostaining was observed.

**Table 2:** Quantification of rhodopsin immunostaining on untreated and treated *rd1* flat-mount retinas.

Treatment	Tissue Fl/Bkgd Fl	Standard deviation	Number of retinas
None	1.36	0.09	6
PBS	1.71	0.17	6
WTS	2.57*	0.44	6
WTSscr25	1.67	0.11	6
WTSscr7	1.63	0.11	6
WTS (PN4)	1.4	0.17	4
WTS (PN4 and PN6)	1.86	0.12	4

*rd1* mice were treated (inontophoresis followed by intravitreal injection) at PN4, PN6, and PN8 (unless otherwise indicated) with a 500 μM of various oligonucleotides (ODNs). Other mice received no treatment (None) or were treated with vehicle (PBS). WTS (PN4) are data from mice treated only at PN4 (e.g., only one treatment total). WTS (PN4 and PN6) are data from mice treated at PN4 and PN6 (e.g., only two treatments total). At PN28, retinas were harvested, flat-mounted, and assayed for rhodopsin immunoreactivity using a fluorescently labeled secondary antibody. Fluorescence was quantified by using the luminosity feature of Adobe Photoshop to normalize the average brightness level per pixel of the tissue region to that of the background region in each flat-mount image. Data are mean of normalized fluorescence ±standard deviation. Sampling was number of retinas per group. Three separate treatments with WTS produced rhodopsin immunoreactivity significantly greater than other treatments (*p<0.001 by simple analysis of variance (ANOVA) with Student-Newman-Keuls post-hoc testing). All other treatments were statistically indistinguishable from PBS treatment (p>0.05).

**Figure 4:**
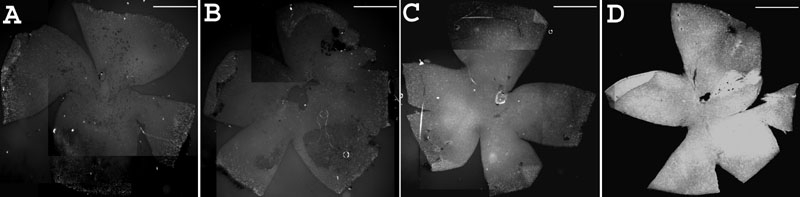
Responsiveness of rhodopsin immunoreactivity to the number of oligonucleotide treatments. **A**: Three treatments with PBS (PN4, PN6, and PN8). **B**: One treatment with ODN at PN4. **C**: Two treatments with ODN (PN4 and PN6). **D**: Three treatments with ODN (PN4, PN6, and PN8). Scale bars : **A**, **B**, **C**, and **D**, 1 mm.

While one single iontophoresis/injection treatment with WTS at PN4 had no effect on photoreceptor survival, two treatments at PN4 and PN6 induced a detectable, though not statistically significant, increase of rhodopsin immunostaining (Table 2, Figure 4). Photoreceptor survival, as evaluated by rhodopsin immunostaining on whole flat-mount retinas, was significantly increased by three successive treatments with 1 μl of the 500 μM WTS ODN. Performing treatments at PN6, PN8, and PN10 provided a rescue similar to that observed when treatments were delivered at PN4, PN6, and PN8 (data not shown).

The application of three successive treatments (iontophoresis prior to injection) at PN4, PN6, and PN8 with 1 μl of 500 μM WTS ODN had a significant effect on rhodopsin expression in PN28 *rd1* mice, indicating increased photoreceptor survival. These conditions were used for all further experiments.

### Rhodopsin immunostaining on eye sections at PN28

**Figure 5:**
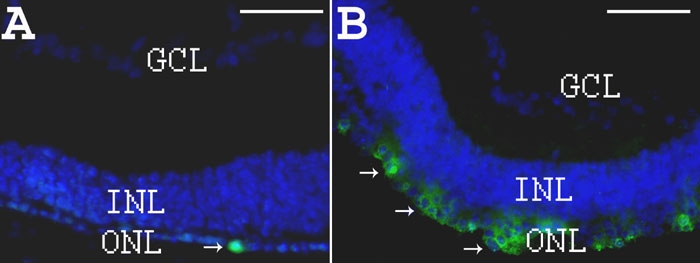
Rhodopsin immunohistochemistry on eye sections from PBS- or oligonucleotide-treated *rd1* mice at PN28. **A**: DAPI staining in blue and rho-4D2 immunostaining in green (arrows) on section from PN28 PBS-treated *rd1* retina. **B**: DAPI staining in blue and rho-4D2 immunostaining in green (arrows) on section from PN28 ODN-treated *rd1* retina. Scale bars are **A** and **B**, 150 μm.

Clusters of rhodopsin positive cells in multiple rows were detected across the retinas of mice treated with WTS ODN (Figure 5B), while some cells in a single row of the residual ONL were labeled in PBS-treated eyes (Figure 5A), demonstrating that treatment by iontophoresis followed by injection of specific ODNs induced the survival of rod photoreceptors at PN28.

### Rhodopsin immunostaining on eye sections at PN33: Confirmatory experiments

**Figure 6:**
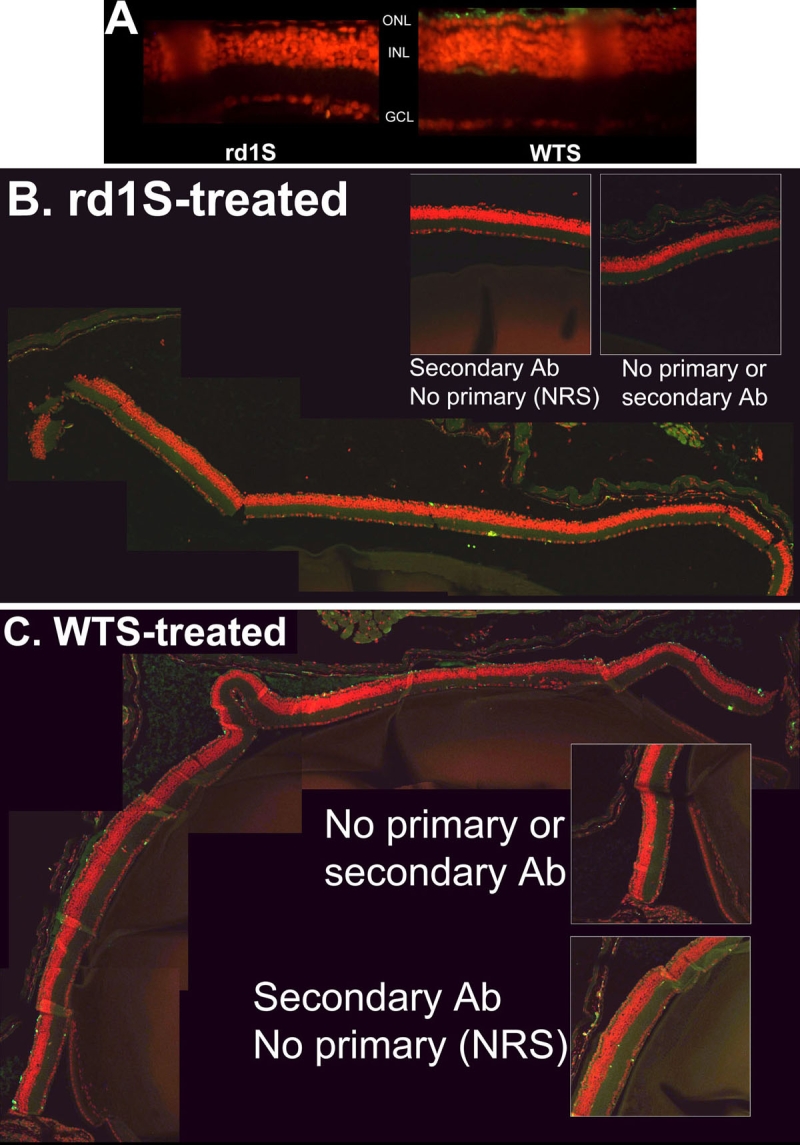
Effect of treatment with WTS versus rd1S at PN33: A confirmatory experiment. In experiments conducted with an independent C3H/henJ colony, littermates treated with WTS ODN had many more rhodopsin-immunopositive cells in the ONL than littermates treated with rd1S, the identical ODN with the exception of having the mutant *rd1* nucleotide at the *rd1* mutation site. Tissue was harvested and sections prepared at PN33. **A**: Fluorescent micrographs of retina sections from rd1S-treated mouse (left panel) and WTS-treated mouse (right panel). Rhodopsin immunosignal is green, counterstained nuclei are red. **B**: Composite image of confocal micrographs of retina section from mouse treated with rd1S. Rhodopsin immunosignal is green, counterstained nuclei are red. Very little rhodopsin signal is apparent. Insets are control sections in which no antibody was used or secondary antibody was used by normal rabbit sera (NRS) was substituted for primary antibody. **C**: Composite image of confocal micrographs of retina section from mouse treated with WTS. Many rhodopsin-positive cells are apparent in the putative photoreceptor layer. Insets are as in **B**. Quantification of rhodopsin-positive cells in photoreceptor layers showed that significantly more signal was observed with WTS treatment compared to rd1S treatment (see "Results").

In experiments conducted with an independent C3H/henJ (*rd1*) colony, littermates treated with WTS ODN had more rhodopsin-immunopositive cells in the ONL than littermates treated with rd1S, the identical ODN with the exception of having the mutant *rd1* nucleotide at the *rd1* mutation site. This effect was observed with either 4D2 (Figure 6A) or 1D4 rhodopsin antibodies (Figure 6B,C). Quantification of the data obtained with 1D4 antibody showed that this difference was statistically significant (WTS-treated: 97±12, N=11; rd1S-treated: 53±15, N=6. p=0.0438 by Student's unpaired t-test). In experiments in which one eye of a littermate was treated with rd1S and the other eye was treated with WTS, such that each mouse was its own control, treatment with WTS again resulted in more rhodopsin-immunopositive cells than treatment with rd1S (45 ±6.4 versus 26 ±11, respectively, four mice per group. p=0.0454 by Student's paired t-test).

### Detection of β-PDE immunoreactivity in retina sections at PN28

**Figure 7:**
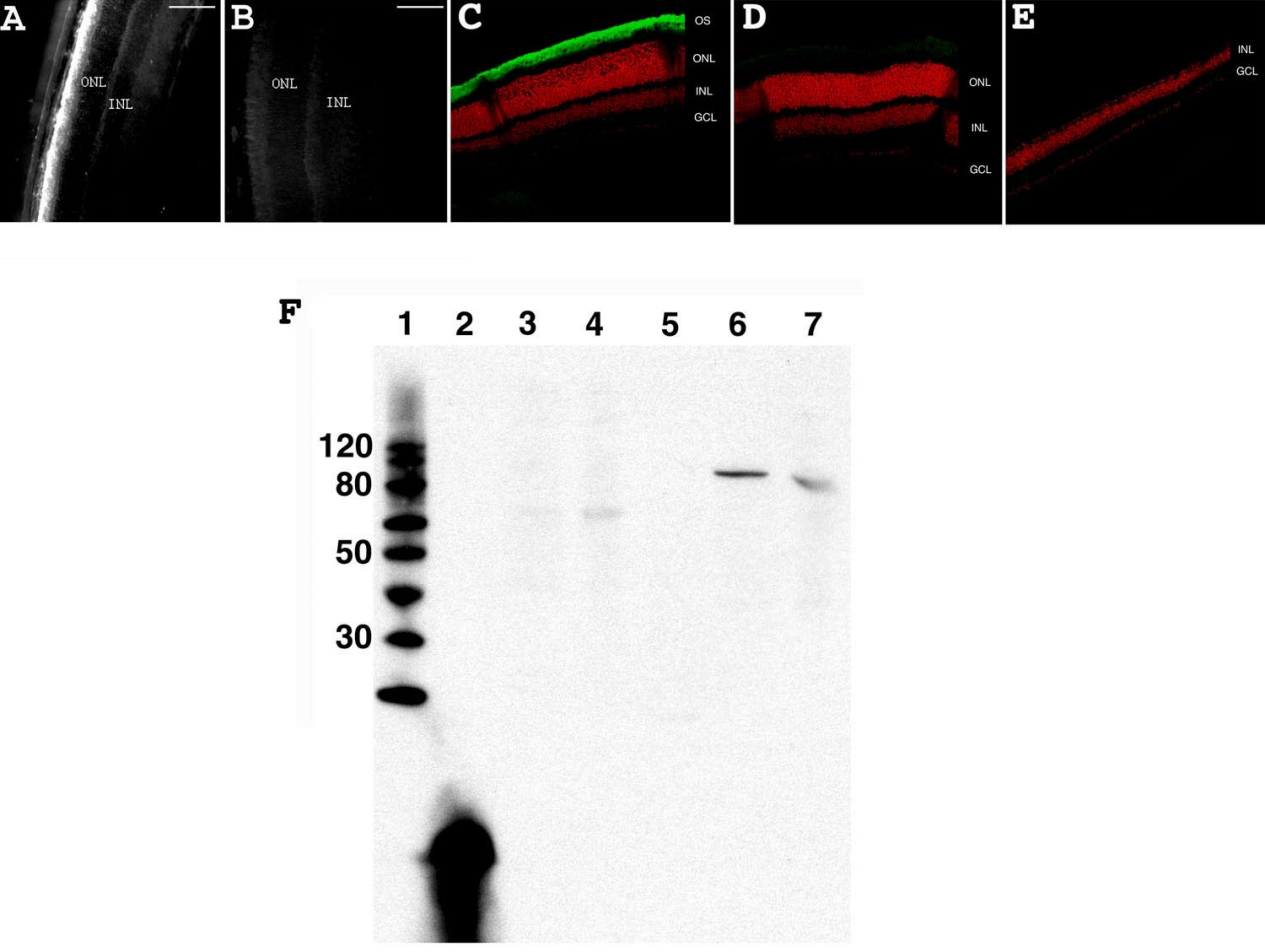
β-PDE immunohistochemistry and western blot. **A**: Wild type +/+ eye section from a mouse at PN28. **B**: Control wild type +/+ eye section from a mouse at PN28 using normal rabbit sera. **C**: Confocal micrograph from a C57/BL6 (wild type; +/+) retina reacted with β-PDE primary antibody and Oregon Green-conjugated goat anti-rabbit IgG (giving green signal in figure) and counter-stained with propidium iodide (giving red signal in figure). **D**: Confocal micrograph from wild type retina reacted with normal rabbit serum. **E**: Confocal micrograph from a C3H/henJ mouse (*rd1*; -/-) retina reacted with β-PDE primary antibody and Oregon green-conjugated goat anti-rabbit IgG. OS indicates outer segments, ONL indicates outer nuclear layer, INL indicates inner nuclear layer, GCL indicates ganglion cell layer. **F**: Anti-β-PDE "western" immunoblot. Lane 1 is the molecular weight marker (sizes given on left), lane 2 is the polypeptide antigen against which the antibody was raised, lane 3 is protein from a C3H (*rd1*) mouse retina, lane 4 is protein from a rd10 mouse retina, lane 5 is protein from an FVB (*rd1*) mouse retina, lane 6 is protein from a CCRC (wild type +/+) mouse retina, lane 7 is protein from a Balb/C (wild type +/+) mouse retina. All mice were at least 30 days old at time of tissue harvest. Scale bars are **A**, and **B**, 100 μm. These data suggest that the antibodies are highly specific and selective to β-PDE.

An antibody directed against β-PDE specifically labeled rod outer segments in the wild type mice at PN28 (Figure 7A-E). The specificity of the anti-β-PDE antibody was confirmed by immunobloting with a specific signal on wild type retinas and an absence of signal on *rd1* retinas at approximately 88-90 kDa (Figure 7F). Some immunoreactivity is observed at about 60 kDa in C3H *rd1* and *rd10* mice. (*rd10* mice do not express β-PDE in adulthood [41].)

**Figure 8:**
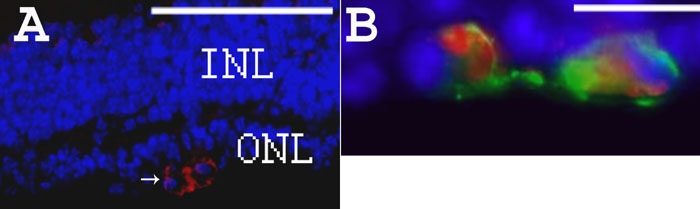
Rhodopsin and β-PDE immunohistochemistry on eye sections from PBS- or WTS ODN-treated *rd1* mice at PN28. **A**: DAPI staining in blue and β-PDE immunostaining in red (arrows) on section from PN28 ODN-treated *rd1* retina. **B**: Combined fluorescence of β-PDE immunostaining in red, rho-4D2 immunostaining in green and DAPI staining in blue on section from PN28 ODN-treated *rd1* retina. Scale bars are **A**, 150 μm; **B**, 10 μm. β-PDE immunoreactivity is associated with cells in the remaining outer nuclear layer (ONL). This staining appears associated with the cytoplasm, not the nucleus. The rhodopsin immunoreactivity is associated with the same cells in the ONL and the immunoreactivity seems more peripheral to the cytosolic β-PDE staining, suggesting a plasma membrane localization of the rhodopsin immunoreactivity. Relatively few cells in the residual ONL exhibit either rhodopsin or β-PDE immunoreactivity at P28 even after three ODN treatments; however, those remaining positive cells exhibit some degree of clustering.

In the *rd1* mouse without ODN treatment, β-PDE immunoreactivity was not present at any stage on retina sections. However, in the 500 μM WTS ODN-treated *rd1* eye sections, a positive fluorescent signal for β-PDE was observed (Figure 8A). An average of 26±6 β-PDE immunopositive cells were detected on whole sections from ODN-treated flat-mount retina (3.5 mm long by 10 μm thick). The 3.5 mm by 10 μm section corresponds to a surface area of 0.035 mm^2^, or 743 positive cells per mm^2^. The estimated surface area of a retina is two-thirds the surface of a sphere (4pr^2^), and for a radius of 1.25 mm at PN28, this is 13.1 mm^2^. The entire retina therefore has roughly 743x13.1=9730 β-PDE positive cells. Double labeling with rho-4D2 showed that cells positive for β-PDE were also positive for rhodopsin (Figure 8B). However, rhodopsin-expressing cells were much more numerous than those expressing β-PDE (Figure 5B and Figure 8A). No significant fluorescence due to β-PDE immunoreactivity was observed when ODN was omitted in otherwise complete treatments (data not shown).

### Analysis of conversion of genomic DNA from *rd1* to wild type

**Figure 9:**
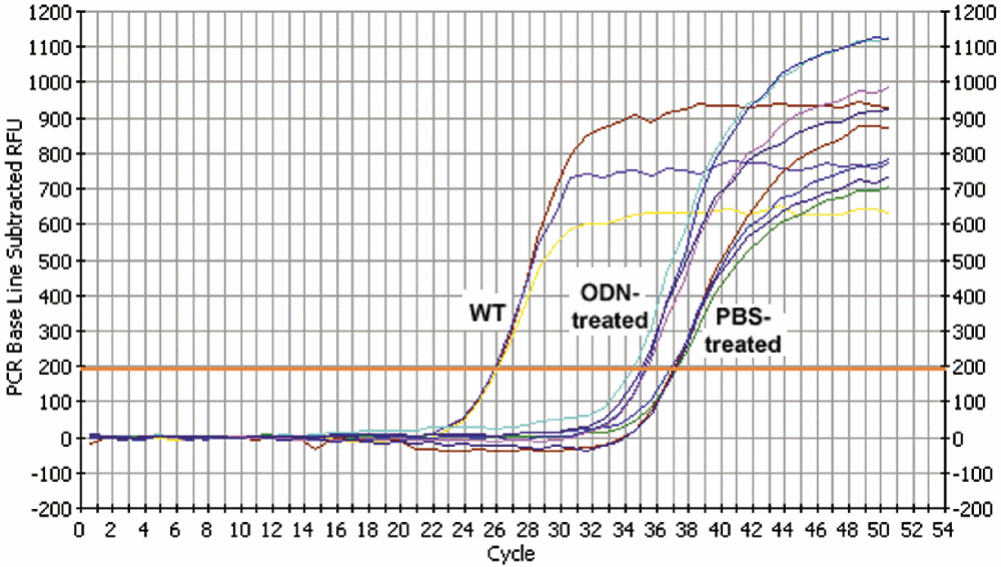
Representative plots of allele specific real time polymerase chain reaction. The graph shows the real-time detection of fluorescence resulting from intercalation of SybrGreen fluorescent dye into double-stranded PCR products. Template DNA was isolated from BALB/c mouse (WT), retinas of *rd1* mice treated with WTS oligonucleotide (ODN-treated), or retinas of *rd1* mice treated with PBS (PBS-treated). Primers were specific for wild type allele. Each curve represents a different eye. Each experimental sample was assayed in 5-10 replicates. See "Materials and Methods" for details of detection threshold calculation (orange horizontal line), normalization, and quantification. Control experiments in which WT ODNs were doped into *rd1* DNA show no shift to the left. Numerous experiments with *rd1* DNAs isolated from many different individual mouse eyes all showed the typical curve of the PBS-treated samples crossing threshold at about 37 cycles.

DNA extracted from ODN- or PBS- treated *rd1* retinas and from wild type retinas was used as DNA template in allele-specific real-time PCR with primers designed to amplify only wild type DNA. The threshold cycle values (C_t_) were 35±0.6 (6 retinas) for the ODN-treated group and 37±0.5 (5 retinas) for the negative control PBS group. The C_t_ for the same amount of pure wild type genomic DNA was 26±0.1 (6 retinas; Figure 9). Treatment of the *rd1* mice, as performed in our study, leads to a significantly lower C_t_ compared to the negative control PBS group (unpaired t-test; p=0.0334), indicating that the ODN-treated group contained wild type DNA and suggesting that treatment induced repair of genomic DNA in *rd1* mice. Based on the efficiency analysis, the effect of treatment on the appearance of wild type β-PDE DNA copy number was taken as 1.95^(a-b)^ where a=C_t_ for ODN treatment and b=C_t_ for wild type genomic DNA. The 1.95^(a-b)^ value divided into 100% provides the percent conversion of the mutant adenine to wild type, i.e., 100/1.95^(a-b)^=100/1.95^(35-26)^=0.2%.

## Discussion

Our results provide evidence that gene repair in photoreceptor cells is feasible when iontophoresis is combined with intravitreal injection of a phosphorothioate single-stranded ODN. β-PDE gene repair was detected in 0.2% of the genomic DNA from treated *rd1* retinas. Phenotypically, this repair was demonstrated by the appearance of β-PDE immunoreactivity and preservation of rhodopsin immunoreactivity in treated *rd1* mice retinas at PN28, a stage when untreated or mock treated mice do not express any β-PDE or significant rhodopsin. Despite the relatively low number of converted copies of genomic DNA, a significant rescue of photoreceptors was observed. These effects were not seen in eyes treated with buffer or with ODNs having the *rd1* mutant sequence, a definitive control for this therapeutic approach. Further, critical experiments were confirmed in two separate laboratories by several different researchers using independent mouse colonies and ODN preparations from independent sources. The present work is a proof-of-concept exercise that demonstrates the feasibility of this non-viral, ODN-targeted gene repair strategy in the neural retina.

For targeted gene repair, the delivery of a sufficient amount of ODNs to the target cells is thought to be critical. In the eye, direct intravitreal injection allowed little ODN to reach photoreceptor nuclei. As shown by the distribution study (Figure 1), the application of iontophoresis prior to the injection of ODNs results in an increased penetration of ODNs into photoreceptor nuclei. Only repeated treatment induced a significant preservation of photoreceptors. Therefore, in our study, photoreceptor cell rescue required repeated delivery and a critical amount of intravitreal ODN combined with an enhanced penetration efficiency to the target cells. Others studies have shown that iontophoresis increases intracellular penetration of small gene fragments [35-37]. It is surprising that, in the present study, iontophoresis before intravitreal injection worked as well as iontophoresis after injection. It is possible that iontophoresis in our experiment was not critical for moving DNA, but instead iontophoresis may boost ODN transport by current-induced changes of the plasma membrane organization or changes in the structure and constituents of extracellular matrices [48]. In the skin, transport during post-iontophoretic periods has been described [49], and the use of saline iontophoresis prior to drug application was previously shown to increase drug penetration [50]. Further experiments will be needed to understand this phenomenon.

The type of ODN is another critical issue in gene repair. In our model, the sense ODN (targeting the transcribed strand of genomic DNA) and antisense ODN (targeting the non-transcribed strand of genomic DNA) induced roughly equivalent genomic repair. Several studies have shown that the strandedness of ODNs influences their targeted repair efficacy [9,23,51]. In a previously reported in vitro study, antisense ODNs were found more effective than sense ODNs for inducing gene repair [8]. However, the superior efficacy of the antisense ODN is not universally observed; recent in vitro and in vivo studies have found the sense ODN to be significantly more effective [23,52-54]. Several factors such as transcription activation, the phase of the cell cycle, and genomic sequences surrounding the target mutation may influence the strand bias [54]. We have developed an in vitro model to study the importance of the target DNA sequence in gene repair. It is based on the introduction of different target sequences at a single identical genomic site in 293T cells. Using this model, we have demonstrated that strand bias is sequence specific. Using this model, we demonstrated that antisense and sense ODNs were roughly equivalent in targeting the sequence containing the *rd1* mutation [25]. Although this in vitro approach was not conducted using retinal cells, it will be useful in assessing what factors potentially influence the frequency of *rd1* correction. The selection of optimized parameters will be applied in vivo to enhance the repair of the *rd1* mutation.

The minor effect of PBS or scrambled ODN treatment on photoreceptor survival as observed on flat-mount retinas rhodopsin immunohistochemistry compared to untreated control (see Table 2) may be attributed to the induction of endogenous neurotrophic factors, known to delay retinal degeneration in the *rd1* mouse model [39,55,56]. Such an effect has been detected in eyes following surgical interventions and other forms of mock, sham, or vehicle control treatments [57-62]. A neurotrophic effect may also explain the much larger number of preserved rhodopsin-positive cells than β-PDE positive cells. Thus, a strategy combining our therapeutic approach with the additional use of neurotrophic factors may potentiate the effect of genomic repair.

Our results are proof of concept. Although the incidence of repair is low, the characteristics of the *rd1* degeneration as a model for human disease should be considered when weighing the potential of this approach. In the *rd1* mouse, retinal degeneration begins soon after birth and progresses quickly, allowing only a narrow window of opportunity for effective gene repair. Furthermore, in this mouse model, an extensive and very rapid degenerative process results in a toxic environment for corrected cells. In humans, most of the degenerative diseases apparently progress slowly, hypothetically allowing a longer therapeutic window. At the present, other models of slowly progressing retinal degeneration are being evaluated in our laboratories. Using these models, we will be able to determine whether applying a larger number of treatments will increase targeted gene repair efficiency and whether gene repair allows for a permanent rescue of photoreceptors.

In conclusion, this study provides a proof of principle for non-viral targeted gene repair in photoreceptors of the *rd1* mouse using a combination of iontophoresis and intraocular injection of specific ODNs and opens new avenues for the treatment of ocular degenerative and blinding eye diseases.

## References

[1] Farber DB Lolley RN Cyclic guanosine monophosphate: elevation in degenerating photoreceptor cells of the C3H mouse retina. Science 1974 186 449 51 4369896 10.1126/science.186.4162.449

[2] Yan W Lewin A Hauswirth W Selective degradation of nonsense beta-phosphodiesterase mRNA in the heterozygous rd mouse. Invest Ophthalmol Vis Sci 1998 39 2529 36 9856762

[3] Farber DB Danciger M Identification of genes causing photoreceptor degenerations leading to blindness. Curr Opin Neurobiol 1997 7 666 73 9384551 10.1016/s0959-4388(97)80087-6

[4] Pittler SJ Baehr W Identification of a nonsense mutation in the rod photoreceptor cGMP phosphodiesterase beta-subunit gene of the rd mouse. Proc Natl Acad Sci USA 1991 88 8322 6 1656438 10.1073/pnas.88.19.8322 PMC52500

[5] McLaughlin ME Ehrhart TL Berson EL Dryja TP Mutation spectrum of the gene encoding the beta subunit of rod phosphodiesterase among patients with autosomal recessive retinitis pigmentosa. Proc Natl Acad Sci USA 1995 92 3249 53 7724547 10.1073/pnas.92.8.3249 PMC42143

[6] McLaughlin ME Sandberg MA Berson EL Dryja TP Recessive mutations in the gene encoding the beta-subunit of rod phosphodiesterase in patients with retinitis pigmentosa. Nat Genet 1993 4 130 4 8394174 10.1038/ng0693-130

[7] Agarwal S Gamper HB Kmiec EB Nucleotide replacement at two sites can be directed by modified single-stranded oligonucleotides in vitro and in vivo. Biomol Eng 2003 20 7 20 12485680 10.1016/s1389-0344(02)00088-6

[8] Liu L Rice MC Kmiec EB In vivo gene repair of point and frameshift mutations directed by chimeric RNA/DNA oligonucleotides and modified single-stranded oligonucleotides. Nucleic Acids Res 2001 29 4238 50 11600713 10.1093/nar/29.20.4238 PMC60207

[9] Liu L Rice MC Drury M Cheng S Gamper H Kmiec EB Strand bias in targeted gene repair is influenced by transcriptional activity. Mol Cell Biol 2002 22 3852 63 Erratum in: Mol Cell Biol. 2003 Aug;2315:5473 11997519 10.1128/MCB.22.11.3852-3863.2002 PMC133839

[10] Wu XS Liu DP Liang CC Prospects of chimeric RNA-DNA oligonucleotides in gene therapy. J Biomed Sci 2001 8 439 45 11702006 10.1007/BF02256605

[11] Cole-Strauss A Yoon K Xiang Y Byrne BC Rice MC Gryn J Holloman WK Kmiec EB Correction of the mutation responsible for sickle cell anemia by an RNA-DNA oligonucleotide. Science 1996 273 1386 9 8703073 10.1126/science.273.5280.1386

[12] Tagalakis AD Graham IR Riddell DR Dickson JG Owen JS Gene correction of the apolipoprotein (Apo) E2 phenotype to wild-type ApoE3 by in situ chimeraplasty. J Biol Chem 2001 276 13226 30 11278248 10.1074/jbc.C000883200

[13] Rando TA Disatnik MH Zhou LZ Rescue of dystrophin expression in mdx mouse muscle by RNA/DNA oligonucleotides. Proc Natl Acad Sci USA 2000 97 5363 8 10805797 10.1073/pnas.97.10.5363 PMC25834

[14] Bartlett RJ Stockinger S Denis MM Bartlett WT Inverardi L Le TT thi Man N, Morris GE, Bogan DJ, Metcalf-Bogan J, Kornegay JN. In vivo targeted repair of a point mutation in the canine dystrophin gene by a chimeric RNA/DNA oligonucleotide. Nat Biotechnol 2000 18 615 22 Erratum in: Nat Biotechnol 2000 Nov;1811:1209 10835598 10.1038/76448

[15] Alexeev V Igoucheva O Domashenko A Cotsarelis G Yoon K Localized in vivo genotypic and phenotypic correction of the albino mutation in skin by RNA-DNA oligonucleotide. Nat Biotechnol 2000 18 43 7 10625389 10.1038/71901

[16] Alexeev V Yoon K Gene correction by RNA-DNA oligonucleotides. Pigment Cell Res 2000 13 72 9 10841028 10.1034/j.1600-0749.2000.130205.x

[17] Kren BT Bandyopadhyay P Steer CJ In vivo site-directed mutagenesis of the factor IX gene by chimeric RNA/DNA oligonucleotides. Nat Med 1998 4 285 90 9500600 10.1038/nm0398-285

[18] Kren BT Parashar B Bandyopadhyay P Chowdhury NR Chowdhury JR Steer CJ Correction of the UDP-glucuronosyltransferase gene defect in the gunn rat model of crigler-najjar syndrome type I with a chimeric oligonucleotide. Proc Natl Acad Sci USA 1999 96 10349 54 10468611 10.1073/pnas.96.18.10349 PMC17891

[19] Kren BT Metz R Kumar R Steer CJ Gene repair using chimeric RNA/DNA oligonucleotides. Semin Liver Dis 1999 19 93 104 10349687 10.1055/s-2007-1007101

[20] Kren BT Chen Z Felsheim R Roy Chowdhury N Roy Chowdhury J Steer CJ Modification of hepatic genomic DNA using RNA/DNA oligonucleotides. Gene Ther 2002 9 686 90 12032688 10.1038/sj.gt.3301762

[21] Bandyopadhyay P Ma X Linehan-Stieers C Kren BT Steer CJ Nucleotide exchange in genomic DNA of rat hepatocytes using RNA/DNA oligonucleotides. Targeted delivery of liposomes and polyethyleneimine to the asialoglycoprotein receptor. J Biol Chem 1999 274 10163 72 10187800 10.1074/jbc.274.15.10163

[22] Bertoni C Rando TA Dystrophin gene repair in mdx muscle precursor cells in vitro and in vivo mediated by RNA-DNA chimeric oligonucleotides. Hum Gene Ther 2002 13 707 18 11936970 10.1089/104303402317322276

[23] Lu IL Lin CY Lin SB Chen ST Yeh LY Yang FY Au LC Correction/mutation of acid alpha-D-glucosidase gene by modified single-stranded oligonucleotides: in vitro and in vivo studies. Gene Ther 2003 10 1910 6 14502220 10.1038/sj.gt.3302096

[24] Nakamura M Ando Y Nagahara S Sano A Ochiya T Maeda S Kawaji T Ogawa M Hirata A Terazaki H Haraoka K Tanihara H Ueda M Uchino M Yamamura K Targeted conversion of the transthyretin gene in vitro and in vivo. Gene Ther 2004 11 838 46 14961068 10.1038/sj.gt.3302228

[25] Andrieu-Soler C Casas M Faussat AM Gandolphe C Doat M Tempe D Giovannangeli C Behar-Cohen F Concordet JP Stable transmission of targeted gene modification using single-stranded oligonucleotides with flanking LNAs. Nucleic Acids Res 2005 33 3733 42 16002788 10.1093/nar/gki686 PMC1174897

[26] Alexeev V Igoucheva O Yoon K Simultaneous targeted alteration of the tyrosinase and c-kit genes by single-stranded oligonucleotides. Gene Ther 2002 9 1667 75 12457280 10.1038/sj.gt.3301862

[27] Taubes G Gene therapy. The strange case of chimeraplasty. Science 2002 298 2116 20 12481116 10.1126/science.298.5601.2116

[28] Sakamoto T Ikeda Y Yonemitsu Y Gene targeting to the retina. Adv Drug Deliv Rev 2001 52 93 102 11672878 10.1016/s0169-409x(01)00191-0

[29] Stodulkova E, Rengarajan K, Takasu I, Martin WD, Padopve SA, Saperstein DA, Nickerson JM, Boatright JH. Initial test oc chimeraplasty in correcting a mouse retinal degeneration. ARVO Annual Meeting; 2001 April 29-May 4; Fort Lauderdale, FL.

[30] Auricchio A Pseudotyped AAV vectors for constitutive and regulated gene expression in the eye. Vision Res 2003 43 913 8 12668060 10.1016/s0042-6989(02)00676-4

[31] Behar-Cohen FF Parel JM Pouliquen Y Thillaye-Goldenberg B Goureau O Heydolph S Courtois Y De Kozak Y Iontophoresis of dexamethasone in the treatment of endotoxin-induced-uveitis in rats. Exp Eye Res 1997 65 533 45 9464186 10.1006/exer.1997.0364

[32] Behar-Cohen FF Savoldelli M Parel JM Goureau O Thillaye-Goldenberg B Courtois Y Pouliquen Y de Kozak Y Reduction of corneal edema in endotoxin-induced uveitis after application of L-NAME as nitric oxide synthase inhibitor in rats by iontophoresis. Invest Ophthalmol Vis Sci 1998 39 897 904 9579469

[33] Behar-Cohen F El Aouni A Le Rouic JF Parel JM Renard G Chauvaud D Iontophoresis: past and future. J Fr Ophtalmol 2001 24 319 27 11285450

[34] Behar-Cohen FF El Aouni A Gautier S David G Davis J Chapon P Parel JM Transscleral Coulomb-controlled iontophoresis of methylprednisolone into the rabbit eye: influence of duration of treatment, current intensity and drug concentration on ocular tissue and fluid levels. Exp Eye Res 2002 74 51 9 11878818 10.1006/exer.2001.1098

[35] Berdugo M Valamanesh F Andrieu C Klein C Benezra D Courtois Y Behar-Cohen F Delivery of antisense oligonucleotide to the cornea by iontophoresis. Antisense Nucleic Acid Drug Dev 2003 13 107 14 12804037 10.1089/108729003321629647

[36] Asahara T Shinomiya K Naito T Shiota H Induction of gene into the rabbit eye by iontophoresis: preliminary report. Jpn J Ophthalmol 2001 45 31 9 11163043 10.1016/s0021-5155(00)00291-4

[37] Voigt M de Kozak Y Halhal M Courtois Y Behar-Cohen F Down-regulation of NOSII gene expression by iontophoresis of anti-sense oligonucleotide in endotoxin-induced uveitis. Biochem Biophys Res Commun 2002 295 336 41 12150953 10.1016/s0006-291x(02)00656-3

[38] Andrieu-Soler C Doat M Halhal M Keller N Jonet L BenEzra D Behar-Cohen F Enhanced oligonucleotide delivery to mouse retinal cells using iontophoresis. Mol Vis 2006 12 1098 107 http://www.molvis.org/molvis/v12/a124/17093395

[39] Frasson M Picaud S Leveillard T Simonutti M Mohand-Said S Dreyfus H Hicks D Sabel J Glial cell line-derived neurotrophic factor induces histologic and functional protection of rod photoreceptors in the rd/rd mouse. Invest Ophthalmol Vis Sci 1999 40 2724 34 10509671

[40] Laird DW Molday RS Evidence against the role of rhodopsin in rod outer segment binding to RPE cells. Invest Ophthalmol Vis Sci 1988 29 419 28 3343097

[41] Chang B Hawes NL Pardue MT German AM Hurd RE Davisson MT Nusinowitz S Rengarajan K Boyd AP Sidney SS Phillips MJ Stewart RE Chaudhury R Nickerson JM Heckenlively JR Boatright JH Two mouse retinal degenerations caused by missense mutations in the beta-subunit of rod cGMP phosphodiesterase gene. Vision Res 2007 47 624 33 17267005 10.1016/j.visres.2006.11.020 PMC2562796

[42] Baehr W Champagne MS Lee AK Pittler SJ Complete cDNA sequences of mouse rod photoreceptor cGMP phosphodiesterase alpha- and beta-subunits, and identification of beta'-, a putative beta-subunit isozyme produced by alternative splicing of the beta-subunit gene. FEBS Lett 1991 278 107 14 1847109 10.1016/0014-5793(91)80095-k PMC5551675

[43] Boatright JH Moring AG McElroy C Phillips MJ Do VT Chang B Hawes NL Boyd AP Sidney SS Stewart RE Minear SC Chaudhury R Ciavatta VT Rodrigues CM Steer CJ Nickerson JM Pardue MT Tool from ancient pharmacopoeia prevents vision loss. Mol Vis 2006 12 1706 14 http://www.molvis.org/molvis/v12/a195/17213800

[44] Molday RS MacKenzie D Monoclonal antibodies to rhodopsin: characterization, cross-reactivity, and application as structural probes. Biochemistry 1983 22 653 60 6188482 10.1021/bi00272a020

[45] Blanks JC Adinolfi AM Lolley RN Photoreceptor degeneration and synaptogenesis in retinal-degenerative (rd) mice. J Comp Neurol 1974 156 95 106 4836657 10.1002/cne.901560108

[46] LaVail MM Sidman RL C57BL-6J mice with inherited retinal degeneration. Arch Ophthalmol 1974 91 394 400 4595403 10.1001/archopht.1974.03900060406015

[47] Sanyal S Bal AK Comparative light and electron microscopic study of retinal histogenesis in normal and rd mutant mice. Z Anat Entwicklungsgesch 1973 142 219 38 4781863 10.1007/BF00519723

[48] Naik A Kalia YN Guy RH Transdermal drug delivery: overcoming the skin's barrier function. Pharm Sci Technol Today 2000 3 318 26 10996573 10.1016/s1461-5347(00)00295-9

[49] Nugroho AK Pasqua OD Danhof M Bouwstra JA Compartmental modeling of transdermal iontophoretic transport: I. In vitro model derivation and application. Pharm Res 2004 21 1974 84 15587918 10.1023/b:pham.0000048187.54125.ac

[50] Drummond PD Prior iontophoresis of saline enhances vasoconstriction to phenylephrine and clonidine in the skin of the human forearm. Br J Clin Pharmacol 2002 54 45 50 12100224 10.1046/j.1365-2125.2002.01597.x PMC1874388

[51] Igoucheva O Alexeev V Yoon K Targeted gene correction by small single-stranded oligonucleotides in mammalian cells. Gene Ther 2001 8 391 9 11313816 10.1038/sj.gt.3301414

[52] Yamamoto T Moerschell RP Wakem LP Ferguson D Sherman F Parameters affecting the frequencies of transformation and co-transformation with synthetic oligonucleotides in yeast. Yeast 1992 8 935 48 1336288 10.1002/yea.320081104

[53] Brachman EE Kmiec EB Targeted nucleotide repair of cyc1 mutations in Saccharomyces cerevisiae directed by modified single-stranded DNA oligonucleotides. Genetics 2003 163 527 38 12618392 10.1093/genetics/163.2.527 PMC1462467

[54] Sorensen CB Krogsdam AM Andersen MS Kristiansen K Bolund L Jensen TG Site-specific strand bias in gene correction using single-stranded oligonucleotides. J Mol Med 2005 83 39 49 Erratum in: J Mol Med. 2005 Jun;836:495-6 15517130 10.1007/s00109-004-0592-6

[55] Cayouette M Gravel C Adenovirus-mediated gene transfer of ciliary neurotrophic factor can prevent photoreceptor degeneration in the retinal degeneration (rd) mouse. Hum Gene Ther 1997 8 423 30 9054517 10.1089/hum.1997.8.4-423

[56] Frasson M Sahel JA Fabre M Simonutti M Dreyfus H Picaud S Retinitis pigmentosa: rod photoreceptor rescue by a calcium-channel blocker in the rd mouse. Nat Med 1999 5 1183 7 10502823 10.1038/13508

[57] LaVail MM Yasumura D Matthes MT Lau-Villacorta C Unoki K Sung CH Steinberg RH Protection of mouse photoreceptors by survival factors in retinal degenerations. Invest Ophthalmol Vis Sci 1998 39 592 602 9501871

[58] Silverman MS Hughes SE Photoreceptor rescue in the RCS rat without pigment epithelium transplantation. Curr Eye Res 1990 9 183 91 2335114 10.3109/02713689008995205

[59] Wahlin KJ Adler R Zack DJ Campochiaro PA Neurotrophic signaling in normal and degenerating rodent retinas. Exp Eye Res 2001 73 693 701 11747369 10.1006/exer.2001.1078

[60] Cao W Wen R Li F Lavail MM Steinberg RH Mechanical injury increases bFGF and CNTF mRNA expression in the mouse retina. Exp Eye Res 1997 65 241 8 9268592 10.1006/exer.1997.0328

[61] Cayouette M Smith SB Becerra SP Gravel C Pigment epithelium-derived factor delays the death of photoreceptors in mouse models of inherited retinal degenerations. Neurobiol Dis 1999 6 523 32 10600408 10.1006/nbdi.1999.0263

[62] Caffe AR Soderpalm AK Holmqvist I van Veen T A combination of CNTF and BDNF rescues rd photoreceptors but changes rod differentiation in the presence of RPE in retinal explants. Invest Ophthalmol Vis Sci 2001 42 275 82 11133879

